# Feasibility of using nitrogen distribution of milk to identify adulterated and reconstituted market milk

**DOI:** 10.5455/javar.2024.k853

**Published:** 2024-12-28

**Authors:** Md. Mehedi Hasan Khandakar, Md. Nasir Sarker, Md. Rezwanul Habib, Md. Sadakatul Bari, Rawnak Jahan, Md. Nurul Islam, Md. Abid Hasan Sarker, Md. Abunaser, Mohammad Ashiqul Islam

**Affiliations:** 1 Department of Dairy Science, Bangladesh Agricultural University, Mymensingh, Bangladesh; 2 Department of Animal Science, Bangladesh Agricultural University, Mymensingh, Bangladesh

**Keywords:** Adulteration, casein, market milk, nitrogen distribution, reconstituted milk, whey protein

## Abstract

**Objective:**

This study aimed to explore the viability of nitrogen distribution in milk to detect adulteration in market milk.

**Materials and Methods:**

Raw cow milk was obtained from the dairy farm at Bangladesh Agricultural University Dairy Farm (BAUDF). Fluid market milk, nonbranded bulk powdered milk, and local brand powdered milk were bought from the Mymensingh city area. The milk samples were T1 (milk from a known source—BAUDF, control group), T2 (reconstituted nonbranded bulk powdered milk), T3 (reconstituted local brand powdered milk), T4 (fluid market milk from Goala), T5 (mixture of 75% T1 and 25% T2), and T6 (mixture of 50% T1 and 50% T2). There were four replications in each variable, and the samples were tested for their physicochemical properties (specific gravity and acidity), gross composition (total solids, ash, milk fat, lactose, and total protein), and nitrogen distribution [casein nitrogen, noncasein nitrogen (NCN), and nonprotein nitrogen (NPN)].

**Results:**

Statistical analysis revealed a significant difference (*p* < 0.05) among the milk samples about their physicochemical properties and gross composition. The T2 and T6 samples imparted lower protein content (*p <* 0.05). Much lower (*p <* 0.05) casein content was found in T2 and T6 than in T1. The NCN content among the samples also differed significantly (*p <* 0.05). All groups showed similar NPN values (*p >* 0.05) but the T1 (*p <* 0.05).

**Conclusion:**

The results from this study show the potential of the nitrogen distribution of milk to detect adulterated and reconstituted market milk; however, a hefty dataset is required before being adopted at the field level.

## Introduction

Long before recorded history, milk was regarded as an ideal meal with unique qualities for human nutrition. Because of its healthful ingredients for all animals, including humans, it is considered a complete meal [1]. Milk production in Bangladesh is rising, but the country still has limitations to cover. In Bangladesh, the annual need for milk is 15.878 MMT, while only 15.04 MMT are produced, and the per-head milk availability is 234.45 ml/day [2]. However, the practical scenario is different as opined by experts in different policy meetings. Eventually, the Bangladesh government imported dairy products, spending $421.273 million in the year 2022–23 [3]. Different types and qualities of powdered milk are available in our country, *i.e.,* from premium quality to nutritionally inferior and, in many cases, contaminated with radioactive materials, heavy metals, and other hazardous materials [4].

Milk powder is the second most susceptible food item to adulteration among the available options. The powdered milk, even with lower grades, is reconstituted and mixed with whole milk and is sold to the market as whole milk. Not only in our country, food adulteration, including milk, is a widespread problem at the food processing and marketing level across the world. In our country, the shortage of milk inspires some unprincipled persons (especially *Goalas*) to make more adulteration in milk by incorporating cheap quality powdered milk, reconstituted milk, urea, rice flour, salt, starch, glucose, vegetable oil, animal fat, melamine, and whey powder to increase the thickness and viscosity of the milk as well as to maintain the composition of the milk.

It should be remembered that water itself is a good adulterant [5]. Due to the higher prevalence of adulteration, it becomes imperative to identify this questioned quality powdered milk as an adulterant in fluid milk because of their possible hazards to human health, such as cancer, blindness, and immunity problems. Various methods have been employed to detect adulterants in milk, e.g., measurement of freezing point, single-frequency conductance analysis, electrical admittance spectroscopy, ultraviolet–visible light spectroscopy, digital image chromatography, and enzyme-linked immunosorbent assay [6]; RNAase activity in milk [7]; β-casein: α-lactalbumin by capillary electrophoresis [8]; and near-infrared spectroscopy [9]. However, all these methods need sophisticated instruments that require fine skill and massive cost involvement.

Literature is also evident that protein fraction analysis could identify adulterants in milk [10] because nitrogen content from outer sources influences protein levels in milk. However, some research has already been done covering the total nitrogen (TN) estimation of milk and milk products in detecting milk adulteration. In our literature search, no research has yet been done focusing on milk’s nitrogen distribution [casein, whey protein, and nonprotein nitrogen (NPN)] to detect adulteration. Hence, the current work was designed to assess the feasibility of using the nitrogen distribution pattern of milk as a tool to identify the adulteration of market milk.

## Materials and Methods

### Experimental site and sample collection

The experiment was carried out at the Dairy Chemistry and Technology Laboratory, Department of Dairy Science, Bangladesh Agricultural University Dairy Farm (BAUDF). Whole milk powder (nonbranded bulk and local brand) was collected from the local market (Chhoto Bazar, Mymensingh, Bangladesh, 24°45'29.9"N 90°24'34.2" E). Raw market milk samples were collected from *Goalas* of different local markets (Bhabakhali Bazar, 24°39'28.8"N 90°27'20.3"E, and Dudhmahal, 24°45'25.7"N 90°24'35.9" E) of Mymensingh Municipality, Bangladesh. A known milk sample was collected from the BAUDF. A total of six groups of milk samples at different adulteration levels were used to compare the distribution of nitrogen among them. There were four replications under one group, and the total number of samples was 24. The milk samples were T1 (milk from BAUDF as a control group), T2 (reconstituted nonbranded bulk powdered milk), T3 (reconstituted local brand powdered milk), T4 (liquid market milk from *Goala*), T5 (mixture of 75% T1 and 25% T2), and T6 (mixture of 50% T1 and 50% T2).

### Reconstituting the powdered milk

In brief, 1 l of reconstituted milk was prepared by dissolving 125 gm of purchased local brand or nonbranded bulk powdered milk with 875 ml of lukewarm water. Rapid powder dissolution was achieved by vigorously shaking the vessel for 20 seconds. After powder dissolution, different samples were prepared by mixing with whole milk with the help of a magnetic stirrer.

### Proximate analysis

A phenolphthalein indicator was used to analyze the samples’ acidity by titration with 0.1-N NaOH, and the specific gravity was calculated from the corrected Quevenne Lactometer reading. Oven drying [J.P. Selecta; S.A. ctra Nil km: 585.1, Abrera (Barcelona), Spain] at 105°C for 24 h was used to obtain the total solid (TS) content of the samples. These dried samples were then initiated at 600°C for 6 h in a muffle furnace (VULCAN A-550, Ney^®^, USA) to measure the ash content. The Babcock method was employed to get the fat content of the samples. The carbohydrates (mainly lactose) and other contents (other remaining substitutes) of different samples were calculated by subtracting the sum of milk fat, total protein, and ash content from the TS content. Carbohydrate and others (%) = TS (%) – [Milk fat (%) + Total protein (%) + Ash (%)]

### Analysis of nitrogen distribution in the samples

The Kjeldahl method was used to assay the TN, noncasein nitrogen (NCN), and NPN content [11]. To determine the nitrogen content of the samples, the milk was first skimmed by centrifugation at 3,500 rpm for 5 min. Each of the nitrogen content values was multiplied by 6.38 to obtain the respective protein value.

### TN estimation

For this purpose, 5 ml of skimmed milk was digested in a Kjeldahl tube by using 20 ml of concentrated H_2_SO_4_, 1–2 gm of catalyst mixture (K_2_SO_4_:CuSO_4_:Se = 100:10:1) for 60 min (or until the contents get clear) at 150°C. After cooling, the content to room temperature, neutralization, and distillation was done using 80 ml of 40% NaOH and 5–7 ml of H_3_BO_3_, followed by titration with 0.1-N HCl using a mixed indicator (methylene blue: methyl red = 2:1; 2–3 drops) until the pink color appears.

### NCN estimation

For NCN separation, 5 ml of milk was used. The sample was tempered to 35°C and then cooled to room temperature. Eight milliliters of acetate buffer (10% V/V acetic acid—0.53 ml + 1 N Na-acetate - 0.53 ml + distilled water—6.94 ml) was added to it, followed by centrifugation at 3,500 rpm for 25 min at room temperature. Three-milliliter supernatants containing NCN were used for the estimation of nitrogen following the method as it was in the TN estimation.

### NPN estimation

The procedure for the separation of the NPN fraction was similar to NCN, except using 10% (W/V) TCA (20 ml) instead of acetate buffer, and 6 ml of supernatant was employed on the Kjeldahl procedure for the nitrogen estimation in the nonprotein fractions.

### Casein nitrogen (CN) estimation

To find the CN, NCN and NPN were subtracted from the TN, and the nitrogen value was multiplied by 6.38 to obtain the casein protein value. The formulas are—CN = TN - (NCN + NPN) and Casein = CN × 6.38.

### Statistical analysis

Data were recorded, analyzed, and visualized to figure out the motif of nitrogen distribution in the known whole milk, reconstituted milk, raw market milk, and whole milk adulterated with reconstituted milk (of nonbranded bulk or local brand powdered milk). To compare the mean, one-way ANOVA was done. Tukey’s HSD as a post hoc test was done for mean separation in case of significant differences among the means. Data are presented as mean ± standard deviation. Minitab version 17 was used for this purpose.

## Result and Discussion

### Gross nutritional quality of different samples

The gross nutritional composition of different milk samples is presented in Table 1. As we see in the table, the highest specific gravity was found in both T2 and T6 samples, which were significantly higher (*p <* 0.05) than the T1, T3, and T5 samples, followed by the T4 milk sample from *Goala*. The highest specific gravity in T2 may result in negligible fat content, and T6 might be for using a 50% T2 milk sample. Memon et al. [12] reported that the specific gravity of milk was increased when reconstituted skim milk was added to it. On the other hand, the lowest specific gravity in the T4 group may cause the consequence of the addition of water to market milk [13]. However, the normal range of specific gravity for cow milk samples is 1.027–1.035 with an average of 1.032 [14].

The acidity of the T1, T3, and T4 groups was significantly higher (*p <* 0.05) than other groups, where the lowest value was found in the T2 milk sample. More than half of the normal acidity of whole milk is due to the presence of casein, which may make a difference in the acidity of milk samples. Hence, the lowest acidity in the T2 milk sample might be the result of the least protein content, more specifically the nominal casein content of this group. On the other hand, the highest acidity content of the T4 group might be the result of a prolonged storage time during transportation and the development of lactic acid content due to the proliferation of lactic acid bacteria during the storage period [15].

The TS found in T1, T4, T5, and T6 milk samples was almost similar, ranging from 12.04% to 12.44%. On the other hand, T2 and T3 milk samples contained lower TS content of 11.63% and 11.43%, respectively. Likely, significant differences (*p <* 0.05) existed among the groups for their ash contents. The lowest ash content was measured in the T4 (0.66%), which was statistically minimal compared to the T1 and T2 samples. The highest ash content of the T2 (0.79%) is owing to reconstituted milk from low-quality powdered milk, probably due to the presence of minerals with heavy metals in that sample. Free grazing of the animals on bushes or forages cultivated on saline salts could also lead to the high ash content of milk from those animals [16]. In addition, greater consumption of green grass and lower consumption of concentrate and advanced lactation [17] are probable reasons for higher ash content in milk.

**Table 1.** Gross nutritional composition of different milk samples (mean ± SD).

Parameters	T1	T2	T3	T4	T5	T6	*p*-value	
Specific gravity	1.028^b^ ± 0.00	1.031^a^ ± 0.00	1.028^b^ ± 0.00	1.026^c^ ± 0.00	1.028^b^ ± 0.00	1.031^a^ ± 0.00		0.000
Acidity (%)	0.17^ab^ ± 0.00	0.06^d^ ± 0.01	0.15^b^ ± 0.01	0.18^a^ ± 0.01	0.12^c^ ± 0.01	0.10^c^ ± 0.01		0.000
Total solids (%)	12.23^ab^ ± 0.17	11.63^c^ ± 0.12	11.43^c^ ± 0.06	12.04^b^ ± 0.02	12.44^a^ ± 0.18	12.16^ab^ ± 0.15		0.000
Ash (%)	0.72^ab^ ± 0.10	0.79^a^ ± 0.01	0.77^ab^ ± 0.01	0.66^b^ ± 0.03	0.68^ab^ ± 0.02	0.73^ab^ ± 0.02		0.021
Milk fat (%)	3.77^a^ ± 0.59	0.63^c^ ± 0.06	2.03^b^ ± 0.06	3.07^a^ ± 0.12	3.40^a^ ± 0.10	3.33^a^ ± 0.15		0.000
Protein (%)	3.24^a^ ± 0.43	1.39^c^ ± 0.08	3.19^a^ ± 0.03	3.32^a^ ± 0.08	3.10^a^ ± 0.03	2.37^b^ ± 0.03		0.000
Carbohydrate & Others (%)	4.51^c^ ± 0.85	9.32^a^ ± 0.14	5.44^bc^ ± 0.05	4.99^bc^ ± 0.16	5.26^bc^ ± 0.21	5.27^b^ ± 0.19		0.000

^abcd^Mean values in a row with uncommon superscript letters differed significantly. T1, Control (known source); T2, Reconstituted low quality powdered milk; T3, Reconstituted local brand powdered milk; T4, Market milk from *Goala*; T5, 75% T1 + 25% T2; T6, 50% T1 + 50% T2.

The highest milk fat content was found in the control group (T1, 3.77%), and the lowest was in reconstituted low-quality powdered milk samples (T2, 0.63%) in Table 1. The fat content of the T1, T4, T5, and T6 samples was statistically higher (*p <* 0.05) and ranged from 3.07% to 3.44% but was higher than the T3 group. The imported low-quality skim milk powder and whey powder are not fully safe for human consumption [18], and these poor products are sold in the market as whole milk powder and sometimes mixed with whole milk powder. These low-quality powdered kinds of milk contained the least or negligible average fat content. Closely, the reconstituted milk from the local brand powder milk (T3) did not also meet the minimum standard of milk fat content for whole milk powder. The minimum requirements of milk fat are 3.25%, 3.5%, 3.25%, 3.2%, and 3.5%, according to the Food and Drug Administration, European Union, Codex Alimentarius Commission, Food Safety and Standards Authority, and Bangladesh Standards and Testing Institution, respectively. In this study, T2, T3, and T4 could not satisfy the minimum fat percent requirement.

Table 1 again depicts that there was a significant difference (*p <* 0.05) among the groups for their protein contents. T1, T3, T4, and T5 groups contained significantly higher (*p <* 0.05) protein content ranging from 3.10% to 3.32% than T2 (1.39%) and T6 groups (2.37%). Significantly lower (*p <* 0.05) protein content in T2 and T6 samples was imparted from the low-quality powdered milk containing less fat. While the average protein content in cow’s milk is reported to be 3.32% [19]. Protein content can be influenced by various factors, such as breed, nutrition, parity, stage of lactation, environmental temperature, and diseases [20]. Probably, the main reason for negligible protein content in the T2 group was that it did not contain enough casein protein. Hence, the total protein content was also noticeably below the average value. The T6 milk sample was formulated using 50% reconstituted low-quality powdered milk, which resulted in a lower value than the standard for protein content in market milk.

The carbohydrate content of reconstituted low-quality powder milk samples (T2, 9.32%) was significantly higher (*p <* 0.05) than other milk samples. Contrarily, the control group contained the lowest carbohydrate content (4.51%). The average lactose (milk sugar, the main carbohydrate) content of cow milk is 4.8%, which is close to the control sample. However, the carbohydrate content of T1, T3, T4, T5, and T6 was close to the finding of Islam et al. [21], who reported average milk carbohydrate content of 4.5% and 3.87% in the milk of the Mymensingh area of Bangladesh. The carbohydrate content of the T2 group might increase due to the adulteration of low-quality powdered milk with different components such as flour or sugar. We calculated the carbohydrate content by using a simple calculation formula, and the T2 group showed dramatically higher lactose content due to the least or minimal fat and protein content in the samples of that group.

**Figure 1.**
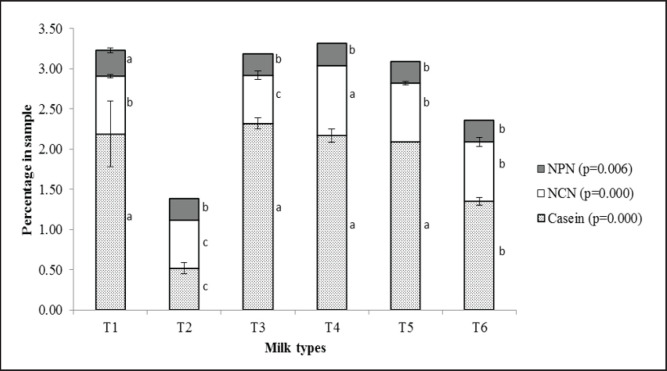
Protein distribution pattern of different milk samples. NPN, nonprotein nitrogen; NCN, noncasein nitrogen; T1, control (known source); T2, reconstituted low quality powder milk; T3, reconstituted local brand powder milk; T4, market milk from *Goala*; T5, 75% T1 + 25% T2; and T6, 50% T1 + 50% T2. ^abc^Mean values in the same patterned bar with uncommon superscript letters differed significantly.

### Protein distribution pattern

Figure 1 exhibits the protein distribution pattern of different graded milk samples. The N-containing portions (TN) in milk are broadly composed of CN, whey NPN or NCN, and NPN. The T4 sample from *Goala* entailed maximum protein content, which was 1.4 times higher than the T6 sample and 2.4 times higher than the T2 sample. A similar trend was found in the case of the casein content of different milk samples. The T3 group contained the highest casein protein, which was 0.97% greater (*p <* 0.05) than the T6 group and 1.8% higher (*p <* 0.05) than the T2 group. Milk samples from the T1, T3, T4, and T5 groups showed close (*p >* 0.05) values for casein protein ranging from 2.09% to 2.32%, and these are significantly higher than the T6 group, followed by the T2 sample (0.5%) that contained the significantly lowest (*p <* 0.05) casein content. The normal range for casein protein in cow milk is 2.46%–2.80% [22].

The casein content of milk could be influenced by using preservatives or storage time [23]. In our study, the probable reason for the low casein content in the T2 group was that the milk powder used for the reconstituted milk might contain higher solids with low protein content. Therefore, lower casein protein content is also reflected in the T6 group. On the other hand, the highest NCN content was found in market milk from *Goala* (T4, 0.8%) and the smallest in reconstituted low-quality powder milk (T2, 0.6%) and reconstituted milk prepared from local brand powdered milk (T3, 0.6%). In our study, the NCN ranged from 0.60% to 0.87% in the case of all samples. Though the NCN contents were within the normal range, there was a significant difference among the groups (*p <* 0.05). The NCN content of T4 samples was significantly (*p <* 0.05) higher, and T2 and T3 samples were significantly lower (*p <* 0.05) than other samples. Higher storage time could reduce the NCN content of milk [24]. The NPN content in different milk samples was almost similar, entailing the lowest (0.27%) in reconstituted local brand powder milk (T3) and the highest (0.32%) in the control group (T1). The NPN content of the T1 sample (0.3%) was statistically higher (*p <* 0.05) than all other groups. The NPN content of tested samples was slightly higher than that of their standard value of 0.10%–0.19% [24]. The NPN content of milk is affected by temperature. Li et al. [25] reported higher NPN content in hot than cold weather. The hot tropical weather of Bangladesh might have contributed to the higher NPN content.

**Figure 2.**
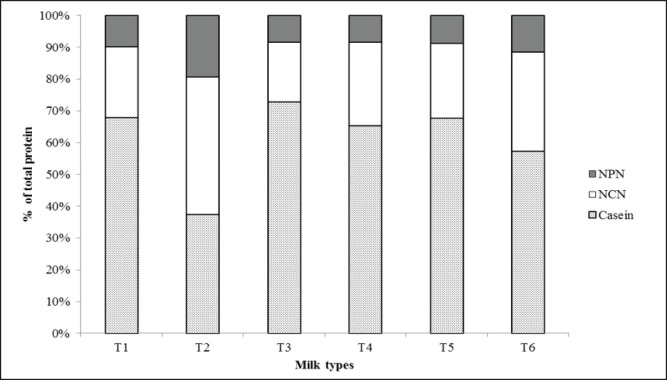
Share of protein fractions to total protein in different milk samples. NPN, nonprotein nitrogen; NCN, noncasein nitrogen; T1, control (known source); T2, reconstituted low-quality powder milk; T3, reconstituted local brand powder milk; T4, market milk from *Goala*; T5, 75% T1 + 25% T2; and T6, 50% T1 + 50% T2.

Figure 2 illustrates the average percentage of casein protein, NCN, and NPN in the total protein of different milk samples. According to Fox and McSweeney [26], the share of casein protein, NCN, and NPN to the total protein of cow milk is around 78%, 17%, and ≈5%, respectively. All the groups showed similar casein protein percentages with the standard value except T2 and T6 (41% and 21% less than the standard average value, respectively). Contrarily, the T2 and T6 groups had relatively higher NCN (26% and 14% higher than the standard value, respectively) and NPN (around 14% higher for T2 and 6% higher for T6) contents than the standard mean values, whereas other groups contained similar percentages. These results indicate that low-quality powdered milk might be adulterated with low-protein ingredients.

## Conclusion

The study showed that the specific gravity, acidity, and gross composition of milk can tell the difference between reconstituted milk, raw milk that has been mixed with reconstituted milk (using nonbranded bulk milk powder), and local brand powder milk reconstitution. By using the casein content, we were able to differentiate reconstituted milk (from nonbranded bulk powdered milk) and raw milk added with 50% fluid milk made from nonbranded bulk milk powder from raw cow milk*. *We also observed differences between the reconstituted milk from local brands of powdered milk and the Goalas milk supply. The NCN content was able to distinguish both the powdered milk from the raw milk. The results on NPN were also found to be variable in different samples. The addition of 25% reconstituted nonbranded bulk milk powder with the raw milk made very little detectable change. From this point forward, this technique could be used to form a national milk databank regarding the protein/nitrogen distribution pattern of milk and may be used in milk quality control, especially regarding adulteration.

## List of abbreviations

ANOVA, Analysis of variance; BAURES, Bangladesh Agricultural University Research System; CE, Capillary electrophoresis; CN, Casein nitrogen; et alia; e.g., exempli gratia; h, hour; HSD, Honestly significant difference; mL, Milliliter; MMT, Million metric ton; NCN, Noncasein nitrogen; NPN, Noprotein nitrogen; RMP, Revolutions per minute; RNAase, Ribonuclease; TN, Total nitrogen; TS, Total solids; UV, Ultraviolet; *Viz.*, Videre licet; et al.; W/V, Weight in volume.

## References

[1] Lambrini K, Aikaterini F, Konstantinos K, Christos I, Ioanna PV, Areti T (2021). Milk nutritional composition and its role in human health. J Pharm Pharmacol.

[2] DLS Livestock economy at a glance, Department of Livestock Services, Dhaka, Bangladesh, 2023–2024. https://dls.portal.gov.bd/sites/default/files/files/dls.portal.gov.bd/page/ee5f4621_fa3a_40ac_8bd9_898fb8ee4700/2024-08-13-10-26-93cb11d540e3f853de9848587fa3c81e.pdf.

[3] Bangladesh Bank Annual import payments of goods and services 2022–2023, 2022–2023. https://www.bb.org.bd/pub/annual/imppayment/imp_2022-23.pdf.

[4] Soomro AA, Khaskheli M, Memon MA, Barham GS, Haq IU, Fazlani SN (2014). Study on adulteration and composition of milk sold at Badin. Int J Res Appl Nat Soc Sci.

[5] Karmaker A, Das PC, Iqbal A (2020). Quality assessment of different commercial and local milk available in the local markets of selected area of Bangladesh. J Adv Vet Anim Res.

[6] Ionescu AD, Cîrîc AI, Begea M (2023). A review of milk frauds and adulterations from a technological perspective. Appl Sci.

[7] Villa C, Costa J, Oliveira MB, Mafra I (2020). Cow’s milk allergens:screening gene markers for the detection of milk ingredients in complex meat products. Food Control.

[8] Ghafoori Z, Tehrani T, Pont L, Benavente F (2022). Separation and characterization of bovine milk proteins by capillary electrophoresis-mass spectrometry. J Separat Sci.

[9] dos Santos Pereira EV, de Sousa Fernandes DD, de Araújo MC, Diniz PH, Maciel MI (2020). Simultaneous determination of goat milk adulteration with cow milk and their fat and protein contents using NIR spectroscopy and PLS algorithms. LWT.

[10] Ma L, Yang Y, Chen J, Wang J, Bu D (2017). A rapid analytical method of major milk proteins by reversed-phase high-performance liquid chromatography. Anim Sci J.

[11] Islam MA, Alam MK, Islam MN, Khan MAS, Ekeberg D, Rukke EO (2014). Principal milk components in Buffalo, Holstein cross, indigenous cattle and Red Chittagong Cattle from Bangladesh. Asian-Australas J Anim Sci.

[12] Memon MA, Khaskheli M, Kamboh AA, Soomro NA, Mangsi AS, Barham GS (2018). Surveillance of milk adulteration and its influence on physico-chemical characteristics of milk in Hyderabad, Pakistan. J Anim Health Prod.

[13] Burke N, Zacharski KA, Southern M, Hogan P, Ryan MP, Adley CC, Díaz AV, García-Gimeno RM (2018). The dairy industry: process, monitoring, standards, and quality. Descriptive food science.

[14] Rahmawati FD, Juwitaningtyas T (2024). Quality analysis of fresh milk based on specific gravity parameters at CV cita nasional in Central Java, Indonesia. J Novel Eng Sci Technol.

[15] Lu M, Shiau Y, Wong J, Lin R, Kravis H, Blackmon T (2013). Milk spoilage: methods and practices of detecting milk quality. Food Nutr Sci.

[16] Khaskheli M, Arain MA, Chaudhry S, Soomro AH, Qureshi TA (2005). Physico-chemical quality of camel milk. J Agric Soc Sci.

[17] Ali W, Akyol E, Ceyhan A, Dilawar S, Firdous A, Qasim MZ (2019). Milk production and composition in camel and its beneficial uses: a review. Turk J Agric Food Sci Technol.

[18] Hamid MA, Siddiky MNA, Rahman MA, Hossain KM (2016). Scopes and opportunities of buffalo farming in Bangladesh: a review. SAARC J Agric.

[19] Yoganandi J, Mehta BM, Wadhwani KN, Darji VB, Aparnathi KD (2014). Evaluation and comparison of camel milk with cow milk and buffalo milk for gross composition. J Camel Pract Res.

[20] Michaelidou AM (2008). Factors influencing nutritional and health profile of milk and milk products. Small Rum Res.

[21] Islam MA, Rashid MH, Kajal MFI, Alam MS (2013). Quality of milk available at local markets of Muktagacha upazila in Mymensingh district. J Bangl Agric Univ.

[22] Guo HY, Pang K, Zhang XY, Zhao L, Chen SW, Dong ML (2007). Composition, physiochemical properties, nitrogen fraction distribution, and amino acid profile of donkey milk. J Dairy Sci.

[23] Vigolo V, Niero G, Penasa M, De Marchi M (2022). Effects of preservative, storage time, and temperature of analysis on detailed milk protein composition determined by reversed-phase high-performance liquid chromatography. J Dairy Sci.

[24] Di Marzo L, Pranata J, Barbano DM (2021). Measurement of casein in milk by Kjeldahl and sodium dodecyl sulfate–polyacrylamide gel electrophoresis. J Dairy Sci.

[25] Li H, Ma Y, Li Q, Wang J, Cheng J, Xue J (2011). The chemical composition and nitrogen distribution of Chinese yak (Maiwa) milk. Int J Mol Sci.

[26] Fox PF, McSweeney PLH (2003). Advanced dairy chemistry-1 proteins.

